# Intracoronary near-infrared spectroscopy: an overview of the technology, histologic validation, and clinical applications

**DOI:** 10.21542/gcsp.2016.18

**Published:** 2016-06-30

**Authors:** Andrew O’Brien, Andrew LaCombe, Aubrey Stickland, Ryan D. Madder

**Affiliations:** Michigan State University College of Human Medicine, Grand Rapids, Michigan, USA; Frederik Meijer Heart & Vascular Institute, Spectrum Health, Grand Rapids, Michigan, USA

## Abstract

Intracoronary near-infrared spectroscopy (NIRS) imaging, which is now clinically available in a combined NIRS and intravascular ultrasound catheter, is a novel catheter-based imaging modality capable of identifying lipid core plaque within the coronary arteries of living patients. The present manuscript provides an overview of intracoronary NIRS imaging with a focus on several concepts essential to individuals seeking to better understand this novel imaging modality. One of the major assets of NIRS is that it has been rigorously validated against the gold standard of histopathology and has been shown to accurately identify histologically-proven fibroatheroma. Clinical studies of NIRS have demonstrated its ability to accurately identify large lipid core plaques at culprit lesions across the spectrum of acute coronary syndromes. NIRS has also been shown to detect lesions at increased risk of causing peri-procedural myocardial infarction during PCI. With regards to predicting future risk, NIRS is seemingly capable of identifying vulnerable patients at increased risk of experiencing subsequent patient-level cardiovascular events. In addition to these clinical applications of NIRS, there are several large prospective observational studies underway to determine if NIRS imaging will be able to identify vulnerable plaques at increased risk of triggering site-specific future coronary events. These studies, once completed, are anticipated to provide valuable data regarding the ability of NIRS imaging to identify plaque vulnerability.

## Introduction

Near-infrared spectroscopy (NIRS) is a widely used technique in analytical chemistry to identify organic substances. NIRS can be simplified into three basic components: (1) a light source; (2) a light detector; and (3) a computer capable of deciphering exiting light into clinically relevant information^1^. NIRS utilizes the attenuation, scatter, and absorption of light emitted at wavelengths of 700–1000 nm to detect a unique optical signature produced by any given unknown molecule^2^. NIRS was first applied *in vivo* in 1977 by Jobsis *et al* who devised a method to monitor oxygenation by detecting the particular optical signature of oxygenated and de-oxygenated hemoglobin via NIRS^3^. Since that time, NIRS has been described in a multitude of other *in vivo* applications, including the recent application of NIRS to detect lipid core plaque (LCP) in the coronary arteries of living patients. Although intracoronary NIRS imaging has been recently reviewed in detail elsewhere^4–6^, we provide an overview of intracoronary NIRS imaging with a focus on three aspects essential to individuals seeking to better understand this novel imaging modality: (1) NIRS technology and its interpretation; (2) studies validating NIRS findings against the gold standard of histopathology; and (3) clinical applications of NIRS imaging in contemporary practice.

## Overview of NIRS technology

### Intracoronary NIRS imaging device

The intracoronary NIRS imaging catheter (TVC Insight Catheter, Infraredx, Burlington, Massachusetts) has applied the same principles of NIRS commonly used in analytic chemistry to identify lipid-rich coronary plaques *in vivo*. The current NIRS imaging system enacts a dual-modality catheter that houses both NIRS and intravascular ultrasound (IVUS) technologies to provide the user with information on both the composition and structure of plaques within the coronary arteries^7–9^. Using a 6 French or larger guide catheter, a combined NIRS-IVUS catheter is manually advanced into a coronary artery over a 0.014 inch guidewire. By pressing a start button on the device, the operator initiates an automated pullback and the catheter scans the target artery at a rate of 5 mm per second^7^. During the automated catheter pullback, more than 30,000 NIRS measurements are obtained within each 100 mm segment of vessel that is imaged^7^. Upon completion of the automated pullback, a NIRS “chemogram” is generated and displayed on the imaging console.

### The NIRS chemogram, block chemogram, and lipid burdens

#### NIRS chemogram

**Figure 1.**
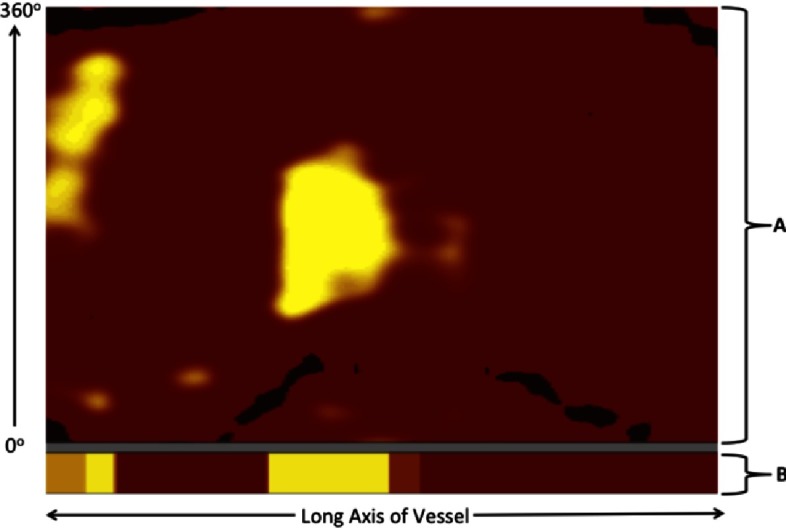
Intracoronary NIRS chemogram. An example of a NIRS chemogram (A) and a corresponding block chemogram (B) are shown. The chemogram is oriented such that distal vessel is located on the right side of the image and the proximal vessel is on the left. The x-axis represents the position along the long axis of the vessel (usually shown with a corresponding scale in millimeters) and the y-axis represents the circumferential position from 0 to 360 degrees. Yellow regions of the chemogram indicate the presence of lipid whereas red regions indicate the absence of lipid. In this example, two lipid cores are present, one of which is in the center of the vessel and the other is in the proximal segment of vessel. NIRS = near-infrared spectroscopy.

Following automated pullback of the NIRS catheter within the target artery, acquired spectra are analyzed to determine the probability of LCP presence at each site within the vessel^4–6^. The probability of LCP presence is displayed visually as a chemogram and a block chemogram, which are automatically generated within a few seconds of completing the NIRS pullback in the artery. The chemogram is oriented such that longitudinal position in the long axis of the vessel is depicted on the x-axis and circumferential position from 0–360 degrees around the inside of the vessel is depicted on the y-axis. For locations in the artery in which NIRS spectra indicate a low probability (i.e. less than 0.6) of LCP presence, the corresponding pixels are depicted as red. For locations in the artery in which NIRS spectra indicate a probability of LCP exceeding 0.6, the corresponding pixels are depicted as yellow. An example of a NIRS chemogram and a description of its orientation are provided in Figure 1.

#### NIRS block chemogram

The contemporary NIRS imaging system also provides a block chemogram that can be found beneath the chemogram on the imaging display^4–6^. When the imaging software creates the block chemogram, the imaged vessel is divided longitudinally into contiguous non-overlapping 2mm segments termed blocks. Each 2mm block is assigned one of four colors based upon the 90th percentile probability of all NIRS measurements within that 2mm segment of vessel:

• Red indicates the probability of LCP is <0.57
• Orange indicates the probability of LCP is 0.57–0.83
• Tan indicates the probability of LCP is 0.84–0.97
• Yellow indicates the probability of LCP is 0.98 or greater

An example of a block chemogram is presented in Figure 1.

#### Lipid core burden index

In addition to providing information regarding the probability of LCP presence at any location within an imaged artery, NIRS is also capable of providing a semi-quantitative estimate of the amount of LCP present within any selected region of interest. This estimate of lipid quantity is termed the lipid core burden index (LCBI) and is calculated as the number of yellow pixels divided by the total valid pixels (red plus yellow) in any region of interest multiplied by 1000^4–6^. The LCBI is reported on a 0 to 1000 scale. In clinical studies of NIRS imaging, lipid burden has frequently been reported as the maximum LCBI in any 4mm section of the artery (maxLCBI_4mm_)^10–14^. Because a 4mm section of vessel is rather narrow, the maxLCBI_4mm_ metric can be considered a surrogate of the circumferential extent of a lipid core. Accordingly, a plaque having a maxLCBI_4mm_ of 700 indicates that the lipid core occupies approximately 70% of the circumference of the vessel at that site.

### NIRS validation against histopathology

Histologic evaluation of coronary autopsy specimens represents the gold standard for LCP detection. One of the major strengths of NIRS imaging is that NIRS has been validated against histopathology for the detection of LCP in several studies. In the first study to validate intracoronary NIRS against histology, Gardner *et al* applied a histologic definition of LCP as a fibroatheroma containing lipid core ≥200 µm thick, having a ≥60°circumferential distribution, and having an overlying fibrous cap thickness <450 microns, a definition mandated by the FDA^15^. In this study, the NIRS block chemogram differentiated segments with and without histologically-proven LCP with an area under the receiver-operator characteristic (ROC) curve of 0.80. This study also demonstrated that a tan or yellow block on the NIRS block chemogram identified a histologically-proven LCP with a specificity of 90%. The high specificity of a tan or yellow block on the NIRS block chemogram for the presence of a histologically-proven fibroatheroma has since been confirmed^16^. NIRS positive lesions have also been demonstrated at autopsy to have larger necrotic cores, more inflammatory cells, and thinner fibrous caps compared to NIRS negative lesions^17^.

**Figure 2.**
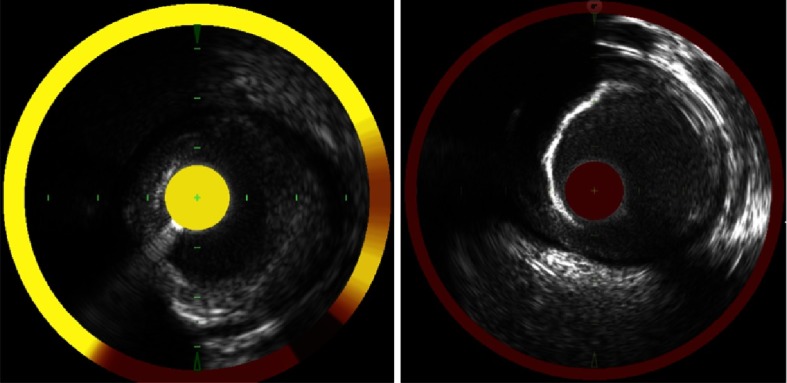
Combined NIRS-IVUS images. The current clinically available NIRS device provides combined automatically co-registered NIRS and IVUS images that are obtained during a single pullback within a coronary artery. Shown are two examples of combined NIRS-IVUS images. In each image the NIRS chemogram is located around the periphery of the greyscale IVUS image, whereas the block chemogram is shown in the central circle. Left: A plaque having a moderate plaque burden by IVUS is composed of lipid as demonstrated by the NIRS chemogram and block chemogram, both of which reveal yellow signal. Right: A calcified plaque by IVUS is shown to be devoid of lipid by NIRS, as both the chemogram and block chemogram are red. IVUS = Intravascular ultrasound; NIRS = near-infrared spectroscopy.

Two autopsy studies have demonstrated the incremental benefit of combined NIRS-IVUS imaging compared to the use of either NIRS or IVUS alone for the identification of histologically-proven fibroatheroma^16,18^. Compared to superficial attenuation by IVUS, Kang *et al* demonstrated that a tan or yellow block on the NIRS block chemogram was associated with a similarly high specificity, but a greater sensitivity for fibroatheroma detection^16^. Importantly, the combination of the NIRS and IVUS findings increased the sensitivity of fibroatheroma detection and improved the positive predictive value compared to ether imaging modality alone^16^. Puri *et al* demonstrated that plaque burden by IVUS and LCBI by NIRS differentiated coronary segments with and without histologically-proven fibroatheroma with similar accuracy^18^. However when plaque burden and LCBI were used together, these combined NIRS-IVUS findings resulted in significant improvement in the accuracy of fibroatheroma detection and were associated with a net reclassification index of 43%. Examples of combined NIRS-IVUS images are provided in Figure 2.

### Clinical application of NIRS imaging

In addition to the use of NIRS imaging in the autopsy studies described above, there are several lines of clinical research that have reported NIRS findings in living patients. In general these NIRS studies have focused on the identification of culprit lesions in patients with acute coronary syndromes (ACS), the detection of lesions at greater risk for periprocedural myocardial infarction during percutaneous coronary intervention (PCI), and the identification of patients at greater risk for future patient-level cardiovascular events. The following sections will describe each of these lines of research in more detail.

### NIRS findings at culprit lesions in patients with ACS

#### STEMI

Post-mortem studies of patients suffering fatal myocardial infarction have shown that most culprit lesions are ruptured thin-capped fibroatheromas with overlying thrombus^19–21^. Considering that an essential component of such thin-capped fibroatheromas at autopsy is a large necrotic lipid core^19–21^, it would be expected that NIRS would likewise identify a large LCP at the culprit site of living patients suffering an acute myocardial infarction.

Among 20 patients presenting with an acute ST-segment elevation myocardial infarction (STEMI), Madder *et al* performed combined NIRS-IVUS imaging in the culprit vessel after Thrombolysis in Myocardial Infarction (TIMI) grade 3 flow was established but prior to stenting^11^. NIRS identified a large, nearly circumferential LCP at the STEMI culprit site in the majority of these patients and STEMI culprit segments were found to have maxLCBI_4mm_ that was 5.8-fold higher than non-culprit segments of the same vessel. Furthermore, the culprit segments had a maxLCBI_4mm_ that was 87-fold higher than segments from an autopsy control group that were known to be free of LCP by histology.

**Figure 3.**
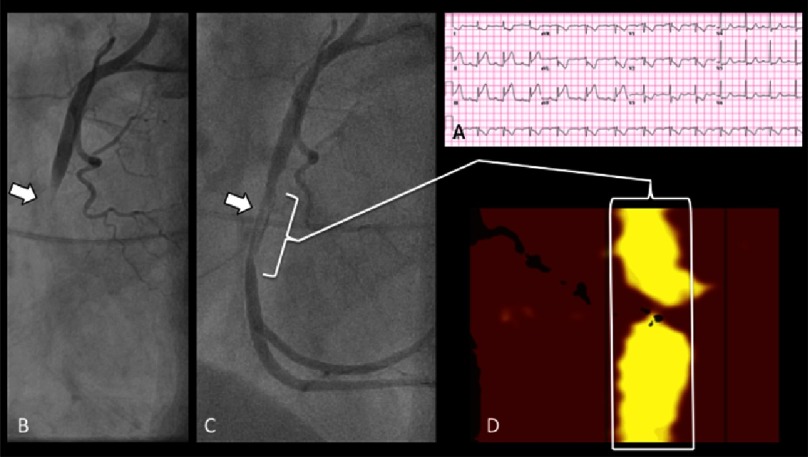
Typical NIRS findings in STEMI. The electrocardiogram of a 54-year-old female presenting with acute chest pain is consistent with an inferior-posterior STEMI (A). The initial angiogram reveals complete occlusion of the mid right coronary artery (arrow, B). Angiography after establishing TIMI grade 3 flow reveals a filling defect consistent with thrombus at the culprit site (arrow, C). NIRS imaging performed prior to stent placement demonstrates a large, nearly circumferential LCP at the culprit site (white bracket, D). LCP = lipid core plaque; NIRS = near-infrared spectroscopy; STEMI = ST-segment elevation myocardial infarction; TIMI = thrombolysis in myocardial infarction.

In this study, a threshold maxLCBI_4mm_ >400 identified STEMI culprit sites with specificity of 98% and a sensitivity of 85%. The significance of this finding is that the LCP generating the maxLCBI_4mm_ >400 signal was likely present in the artery well before the onset of the STEMI. Hence, the maxLCBI_4mm_ >400 signal may be a marker of a vulnerable plaque at increased risk of triggering a future myocardial infarction. A larger, multicenter study confirming these initial NIRS observations in STEMI patients is expected to be published in 2016. An example of typical NIRS findings in a patient with STEMI is provided in Figure 3.

#### Non-STEMI and unstable angina

NIRS evaluations of culprit lesions in patients with non-STEMI and unstable angina have also been performed^12^. Similar to the findings previously reported in STEMI, patients with non-STEMI and unstable angina frequently have a large LCP detected by NIRS at the culprit site that is characterized by a maxLCBI_4mm_ ≥ 400^12^. Accordingly, a threshold maxLCBI_4mm_ ≥ 400 accurately differentiated culprit from nonculprit sites in non-STEMI and unstable angina while retaining the high specificity previously demonstrated in STEMI patients. Interestingly, culprit lesions in non-STEMI more often had a maxLCBI_4mm_ ≥ 400 rather than a moderate (maxLCBI_4mm_ 200–399) or small (maxLCBI_4mm_ <200) lipid burden. In contrast, culprit lesions in unstable angina had lipid burdens that were more evenly distributed among lipid cores that were small, moderate or large. This observation suggests that the clinical presentation of a plaque rupture event may depend not only on the presence of a LCP, but also on the overall burden of lipid at the culprit site^12^. Additional studies are needed to determine how the lipid burden impacts the clinical presentation of a plaque rupture event.

#### Sudden cardiac death

A small series of patients presenting with sudden cardiac death who were successfully resuscitated and subsequently underwent NIRS-IVUS imaging has been published^22^. In this small series of patients, a maxLCBI_4mm_ ≥ 400 was detected by NIRS at each culprit site thought to be responsible for triggering ischemia and the resulting ventricular arrhythmia that lead to cardiac arrest. When considering this observation and those previously demonstrated in STEMI, non-STEMI, and unstable angina, NIRS has now been shown to identify large LCP at culprit sites across the entire spectrum of ACS clinical presentations. A larger study is needed to further evaluate the association between NIRS findings and sudden cardiac death.

### NIRS to predict peri-procedural myocardial infarction

Peri-procedural myocardial infarction remains commonplace, complicating 3–15% of all PCI procedures^13^. One of the pathophysiologic mechanisms thought to account for some instances of peri-procedural myocardial infarction is embolization of lipid-rich material that is mechanically released from a target lesion during angioplasty or stent placement^23,24^. Hence, the identification of lipid at the target lesion prior to PCI may identify those lesions at greater risk of causing PCI-related complications. Although this concept has been demonstrated previously with IVUS^25–27^, optical coherence tomography^28–30^ and non-invasively with computed tomographic angiography^31^, NIRS may offer an advantage over these techniques owing to its automated ability to identify LCP with high chemical specificity.

In an analysis of cases from the COLOR registry, Goldstein *et al* studied target lesions undergoing NIRS imaging prior to PCI and demonstrated that lesions having a maxLCBI_4mm_ ≥ 500 had a 50% rate of peri-procedural myocardial infarction (13). This is in stark contrast to the observation that only 4.2% of target lesions having a maxLCBI_4mm_ <500 were associated with a peri-procedural infarct. In this study target lesions having a maxLCBI_4mm_ ≥ 500 prior to PCI had a 12-fold increased risk of developing a peri-procedural myocardial infarction during PCI^13^.

In the CANARY trial, Stone *et al* confirmed that large LCPs identified by NIRS imaging prior to PCI were significantly associated with a higher risk of peri-procedural myocardial infarction^14^. In this study lesions having a maxLCBI_4mm_ ≥ 600 were associated with peri-procedural myocardial necrosis in 24.7% of cases. However, the CANARY trial, which randomized target lesions having a maxLCBI_4mm_ ≥ 600 to undergo PCI either with or without a distal protection filter, failed to show benefit of distal protection. This finding was perhaps unexpected considering evidence from a prior small study that presented convincing evidence of debris capture using distal protection during PCI on large LCP^32^ and also highlights the uncertainty regarding methods to reduce the risk of peri-procedural complications during PCI of large LCP. It is unknown if other methods shown to reduce the focal lipid burden, such as intensive statin therapy^33^ or mechanical aspiration of lipid content from the target lesion^34^, if performed prior to PCI will reduce the risk of PCI-related complications. Additional study in this area is clearly needed.

### NIRS to identify the vulnerable patient

Whereas the identification of large LCP at heightened risk of triggering peri-procedural myocardial infarction represents one potential clinical use of NIRS imaging, another proposed application of NIRS is to identify patients at increased risk of future patient-level major adverse cardiovascular events. In the ATHEROREMO-NIRS study, investigators performed NIRS imaging within a non-culprit vessel after performing PCI elsewhere in the coronary tree. Patients having an LCBI in the non-culprit vessel above the median value of 43 for the population had a 4-fold increased risk of patient-level cardiovascular events during 1-year of follow up compared to those with an LCBI below the median^35^. Although prior studies have demonstrated a clear association of serum lipid content and cardiovascular risk^36–38^, the observations of the ATHEROREMO-NIRS study suggests that the burden of lipid deposited within coronary tissues, as now detectable by NIRS imaging, also associates with cardiovascular risk. Additional studies examining the ability of NIRS imaging to identify patient-level vulnerability to cardiovascular events are forthcoming^39^.

### NIRS to identify the vulnerable plaque

Given the association of large LCP identified by NIRS with culprit lesions across the spectrum of ACS^10–12,22^ and the propensity of such large LRP to trigger myocardial infarction at the time of PCI^13,14^, it has been proposed that NIRS imaging may be a means to identify vulnerable plaques at increased risk of triggering future site-specific coronary events^4^. Whereas prior studies have demonstrated the ability of IVUS to detect plaques at increased risk of site-specific future events^40–43^, it remains unproven if NIRS findings of a large LRP are in fact at increased risk of triggering future events. There are currently at least two large multicenter prospective observational studies testing the hypothesis that NIRS is capable of identifying vulnerable plaques. These studies include the Lipid Rich Plaque study being performed in the United States and Europe and the PROSPECT II study currently underway in Scandinavia^4^. These studies, once completed, are anticipated to provide valuable data regarding the ability of NIRS imaging to identify plaque vulnerability.

## Conclusion

NIRS imaging, which is now clinically available in a combined NIRS-IVUS catheter, is a novel catheter based imaging modality capable of identifying LCP within the coronary arteries of living patients. One of the major assets of NIRS is that it has been rigorously validated against the gold standard of histopathology and has been shown to accurately identify histologically-proven fibroatheroma. Clinical studies of NIRS have demonstrated its ability to identify culprit lesions across the spectrum of ACS, to detect target lesions at increased risk of causing peri-procedural myocardial infarction, and to identify vulnerable patients. There are several large prospective observational studies underway to determine if NIRS imaging can identify vulnerable plaques.

## Competing Interests

Ryan D. Madder has received research support from Infraredx, Inc. Andrew O’Brien, Andrew Lacombe, and Aubrey Stickland have nothing to disclose.

## References

[1] Wahr JA, Tremper KK, Samra S, Delpy DT (1996). Near-infrared spectroscopy: theory and applications. J Cardiothorac Vasc Anesth.

[2] Delpy DT, Cope M (1997). Quantification in tissue near–infrared spectroscopy. Philos Trans R Soc Lond B Biol Sci.

[3] Jobsis FF (1977). Noninvasive, infrared monitoring of cerebral and myocardial oxygen sufficiency and circulatory parameters. Science.

[4] Madder RD, Stone GW, Erlinge D, Muller JE (2013). The search for vulnerable plaque –the pace quickens. J Invasive Cardiol.

[5] Su J, Grainger SJ, Greiner CA (2015). Detection and structural characterization of lipid-core plaques with intravascular NIRS-IVUSimaging. Interv Cardiol.

[6] Negi SI, Didier R, Ota H (2015). Role of near-infrared spectroscopy in intravascular coronary imaging. Cardiorevasc Revasc Medicine.

[7] Shydo B, Hendricks M, Frazier G (2013). Imaging of plaque composition and structure with the TVC imaging system and TVC insight catheter. J Invasive Cardiol.

[8] Schultz CJ, Serruys PW, van der Ent M (2010). First-in-man clinical use of combined near-infrared spectroscopy and intravascular ultrasound. J Am Coll Cardiol.

[9] Madder RD, Steinberg DH, Anderson RD (2013). Multimodality direct coronary imaging with combined near-infrared spectroscopy and intravascular ultrasound: initial US experience. Catheter Cardiovasc Interv.

[10] Madder RD, Smith JL, Dixon SR, Goldstein JA (2012). Composition of target lesions by near-infrared spectroscopy in patients with acute coronary syndrome versus stable angina. Circ Cardiovasc Interv.

[11] Madder RD, Goldstein JA, Madden SP (2013). Detection by near-infrared spectroscopy of large lipid core plaques at culprit sites in patients with acute ST-segment elevation myocardial infarction. J Am Coll Cardiol Intv.

[12] Madder RD, Husaini M, Davis AT (2015). Detection by near-infrared spectroscopy of large lipid cores at culprit sites in patients with non-ST-segment elevation myocardial infarction and unstable angina. Catheter Cardiovasc Interv.

[13] Goldstein JA, Maini B, Dixon SR (2011). Detection of lipid-core plaques by intracoronary near-infrared spectroscopy identifies high risk of peri-procedural myocardial infarction. Circ Cardiovasc Interv.

[14] Stone GW, Maehara A, Muller JE (2015). Plaque characterization to inform the prediction and prevention of periprocedural myocardial infarction during percutaneous coronary intervention. J Am Coll Cardiol Intv.

[15] Gardner CM, Tan H, Hull EL (2008). Detection of lipid core coronary plaques in autopsy specimens with a novel catheter-based near-infrared spectroscopy system. J Am Coll Cardiol Img.

[16] Kang SJ, Mintz GS, Pu J (2015). Combined IVUS and NIRS detection of fibroatheromas: histopathologic validation in human coronary arteries. J Am Coll Cardiol.

[17] Patel D, Hamamdzic D, Llano R (2013). Subsequent development of fibroatheromas with inflamed fibrous caps can be predicted by intracoronary near-infrared spectroscopy. Arterioscler Thromb Vasc Biol.

[18] Puri R, Madder RD, Madden SP (2015). Near-infrared spectroscopy enhances intravascular ultrasound assessment of vulnerable coronary plaque. Arterioscler Thromb Vasc Biol.

[19] Burke AP, Farb A, Malcom GT (1997). Coronary risk factors and plaque morphology in men with coronary disease who died suddenly. N Engl J Med.

[20] Farb A, Tang AL, Burke AP (1995). Sudden coronary death: frequency of active coronary lesions, inactive coronary lesions, and myocardial infarction. Circulation.

[21] Virmani R, Kolodgie FD, Burk AP (2000). Lessons from sudden coronary death: a comprehensive morphologic classification scheme for atherosclerotic lesions. Arterioscler Thromb Vasc Biol.

[22] Madder RD, Wohns DH, Muller JE (2014). Detection by intracoronary near-infrared spectroscopy of lipid core plaque at culprit sites in survivors of cardiac arrest. J Invasive Cardiol.

[23] Lansky AJ, Stone GW (2010). Periprocedural myocardial infarction: prevalence, prognosis, and prevention. Circ Cardiovasc Interv.

[24] Jaffe R, Charron T, Puley G (2008). Microvascular obstruction and the no-reflow phenomenon after percutaneous coronary intervention. Circulation.

[25] Shiono Y, Kubo T, Tanaka A (2013). Impact of attenuated plaque as detected by intravascular ultrasound on the occurrence of microvascular obstruction after percutaneous coronary intervention in patients with ST-segment elevation myocardial infarction. J Am Coll Cardiol Intv.

[26] Wu X, Mintz GS, Xu K (2011). The relationship between attenuated plaque identified by intravascular ultrasound and no-reflow after stenting in acute myocardial infarction. J Am Coll Cardiol Intv.

[27] Tanaka A, Kawarabayashi T, Nishibori Y (2002). No-reflow phenomenon and lesion morphology in patients with acute myocardial infarction. Circulation.

[28] Lee T, Tonetsu T, Koura K (2011). Impact of plaque morphology assessed by optical coherence tomography on cardiac troponin elevation in patients with elective stent implantation. Circ Cardiovasc Interv.

[29] Tanaka A, Imanishi T, Kitabata H (2009). Lipid-rich plaque and myocardial perfusion after successful stenting in patients with non-ST-segment elevation acute coronary syndrome: an optical coherence tomography study. Eur Heart J.

[30] Yonetsu T, Kakuta T, Lee T (2011). Impact of plaque morphology on creatinine kinase-MB elevation in patients with elective stent implantation. Int J Cardiol.

[31] Kodama T, Kondo T, Oida A (2012). Computed tomographic angiography-verified plaque characteristics and slow-flow phenomenon during percutaneous coronary intervention. J Am Coll Cardiol Intv.

[32] Abdel-karim AR, Papayannis AC, Rangan BV (2011). Stenting of native coronary artery lesions with large lipid core plaques as detected by near-infrared spectroscopy is associated with high frequency of debris retrieval using embolic protection devices. Catheter Cardiovasc Interv.

[33] Kini AS, Baber U, Kovacic JC (2013). Changes in plaque lipid content after short-term intensive versus standard statin therapy: the YELLOW trial. J Am Coll Cardiol.

[34] Erlinge D, Harnek J, Goncalves I (2014). Coronary liposuction during percutaneous coronary intervention: evidence by near-infrared spectroscopy that aspiration reduces culprit lesion lipid content prior to stent placement. Eur Heart J Cardiovasc Imaging.

[35] Oemrawsingh RM, Cheng JM, Garcia-Garcia HM (2014). Near-infrared spectroscopy predicts cardiovascular outcome in patients with coronary artery disease. J Am Coll Cardiol.

[36] Stamler J, Daviglus ML, Garside DB (2000). Relationship of baseline serum cholesterol levels in three large cohorts of younger men to long-term coronary, cardiovascular, and all-cause mortality and to longevity. JAMA.

[37] Boekholdt SM, Hovingh GK, Mora S (2014). Very low levels of atherogenic lipoproteins and the risk for cardiovascular events. J Am Coll Cardiol.

[38] Baigent C, Blackwell L, Emberson J (2010). Efficacy and safety of more intensive lowering of LDL cholesterol: a meta-analysis of data from 170,000 participants in 26 randomised trials. Lancet.

[39] Madder RD, Husaini M, Davis AT (2014). Identification of vulnerable patients by intracoronary near-infrared spectroscopy (abstract). J Am Coll Cardiol.

[40] Stone GW, Maehara A, Lansky AJ (2011). A prospective natural-history study of coronary atherosclerosis. N Engl J Med.

[41] Calvert PA, Obaid DR, O’Sullivan M (2011). Association between IVUS findings and adverse outcomes in patients with coronary artery disease: the VIVA study. J Am Coll Cardiol Imaging.

[42] Stone PH, Saito S, Takahashi S (2012). Prediction of progression of coronary artery disease and clinical outcomes using vascular profiling of endothelial shear stress and arterial plaque characteristics: the PREDICTION study. Circulation.

[43] Cheng JM, Garcia-Garcia HM, de Boer SP (2014). In vivo detection of high-risk coronary plaques by radiofrequency intravascular ultrasound and cardiovascular outcome: results of the ATHEROREMO-IVUS study. Eur Heart J.

